# Anesthetic considerations in an infant with femoral hypoplasia‐unusual facies syndrome and Pierre Robin sequence: A case report

**DOI:** 10.1002/ccr3.7646

**Published:** 2023-07-04

**Authors:** Lauren M. Partyka

**Affiliations:** ^1^ Department of Anesthesiology University of Iowa Iowa City Iowa USA

**Keywords:** anesthetic management, difficult airway, femoral hypoplasia‐unusual facies syndrome, Pierre Robin sequence

## Abstract

**Key Clinical Message:**

Femoral hypoplasia‐unusual facies syndrome is a rare condition of unknown etiology. The phenotype consists of significant femoral hypoplasia with characteristic facial malformations that often overlap with findings seen in patients with Pierre Robin sequence. Anesthesia providers must prepare for difficult intravenous access, difficult airway management, and uncertainties with regional anesthesia.

**Abstract:**

Femoral hypoplasia‐unusual facies syndrome (FHUFS) or femoral facial syndrome is a rare and sporadic condition of unknown etiology. The phenotype consists of significant femoral hypoplasia with characteristic facial malformations that often overlap with findings seen in patients with Pierre Robin sequence. FHUFS is known to cause challenges with anesthesia, including difficulty with endotracheal intubation. Anesthesia providers must be aware of the possible coexistence of FHUFS and Pierre Robin sequence. They need to prepare for difficult intravenous access, difficult airway management, and uncertainties with regional anesthesia.

## 1 INTRODUCTION

Femoral hypoplasia‐unusual facies syndrome (FHUFS) or femoral facial syndrome is a rare congenital syndrome characterized by unilateral or bilateral femoral hypoplasia along with various dysmorphic facial features such as a short nose with a broad tip, a long philtrum, a thin upper lip, upward slanting palpebral fissures, and micrognathia.^1^, ^2^ Thorough physical and airway examination is necessary because FHUFS can also present with Pierre Robin sequence.^3^

The etiology of FHUFS is unknown with a prevalence of <1/1,000,000.^4^ FHUFS mirrors caudal regression syndrome or sirenomelia, which occurs due to insufficient mesoderm in the caudal area of the embryo. Caudal dysplasia results in lumbosacral defects, lower limb dysplasia, and renal agenesis, but it lacks any facial anomalies. A disruption of normal carbohydrate homeostasis during a critical time of embryogenesis may contribute to the development of FHUFS and caudal dysplasia.^1^

Although there are reports of FHUFS with autosomal dominant inheritance, a multifactorial etiology, including fetal constraint secondary to oligohydramnios, is more likely in cases that are sporadic. Maternal diabetes seems to be a risk factor for complex femoral hypoplasia. Up to one‐third of affected individuals have a mother who either had pregestational diabetes or developed it during pregnancy. There may be a possible genetic component in children of non‐diabetic mothers. One case report described a de novo complex chromosome rearrangement of the terminal end of chromosome 2q.^2^ Research to identify a genetic link with the development of FHUFS is ongoing.

With the multiple systemic malformations exhibited in FHUFS, these patients undergo a variety of operations. A recent case report discussed a 42‐year‐old patient with FHUFS who underwent an upper endoscopy procedure. These patients can present to the operating room well into adulthood.^5^ Due to the rarity of the syndrome, an anesthesia provider is unlikely to encounter a patient with FHUFS on a routine basis. However, detailed anesthesia management is seemingly absent from the literature surrounding the care of a patient with FHUFS. There is little information regarding mask ventilation and other intubation equipment outside of a fiberoptic intubation through a supraglottic airway.^6^

We describe a case of an infant with FHUFS and Pierre Robin sequence to provide greater awareness of the potential anesthetic challenges in the care of these patients. The patient's guardian provided written Health Insurance Portability and Accountability Act authorization for this report. This article adheres to the case report checklist guidelines.

## 2 CASE PRESENTATION

A 13‐week‐old (ex‐31‐week gestation) infant presented for a right inguinal hernia repair with orchiopexy and placement of a gastrostomy tube. A notable complication during the pregnancy was premature rupture of membranes at 15 weeks, which led to oligohydramnios. The patient weighed 3.45 kg. The medical history included skeletal dysplasia, cleft palate, undescended testes, bronchopulmonary dysplasia, systemic hypertension on enalapril, retinopathy of prematurity, choroid plexus cysts, and a difficult airway for intubation. At birth, the neonatal intensive care team made four attempts before successfully intubating the newborn. As a result of respiratory failure due to prematurity, pulmonary hypertension, and respiratory distress syndrome, the patient remained intubated for 2 weeks.

**FIGURE 1:**
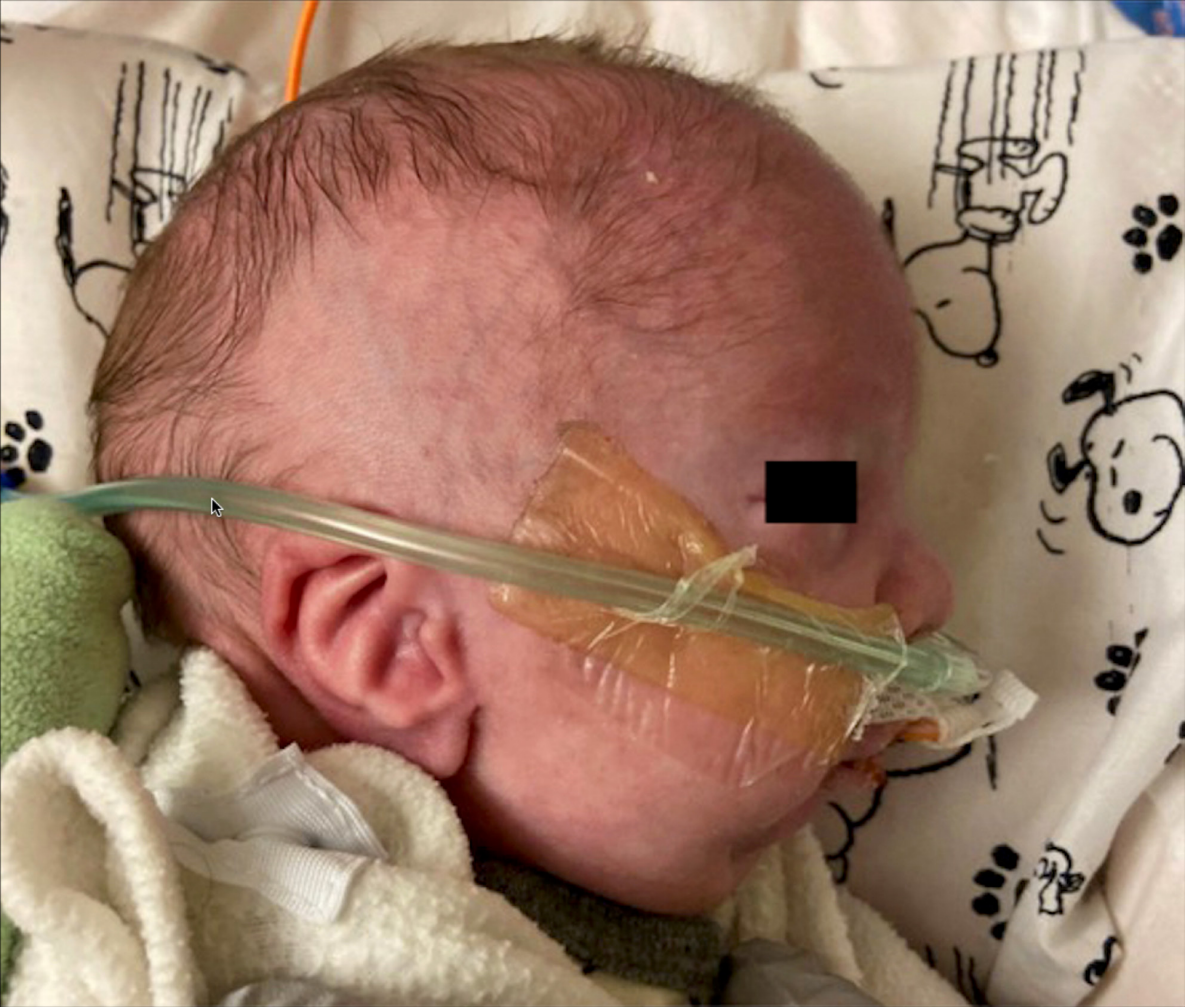
Facial feature present in Pierre Robin sequence: microretrognathia.

During the subsequent evaluation, the chromosomal microarray was negative. The skeletal survey identified bilateral femoral deficiency, bilateral proximal radius and ulna dislocation, bowing of the clavicles, inverted feet, concerning for clubfeet, micrognathia, and fusion of several sacral vertebral bodies. A transthoracic echocardiogram prior to our procedure showed a patent foramen ovale and the resolution of prior pulmonary hypertension. On physical examination, the patient had microretrognathia, a bulbous nose tip, posteriorly rotated low‐set ears (Figure 1), glossoptosis, a cleft soft palate, and an incomplete hard cleft palate consistent with Pierre Robin sequence. The patient was able to breathe and handle secretions best in a side‐lying or prone position, rather than supine. The genetics team subsequently diagnosed the patient with FHUFS given the presence of hypoplastic femurs and multiple abnormal facial characteristics including a cleft palate and low‐set, poorly formed ears.^1^

**FIGURE 2:**
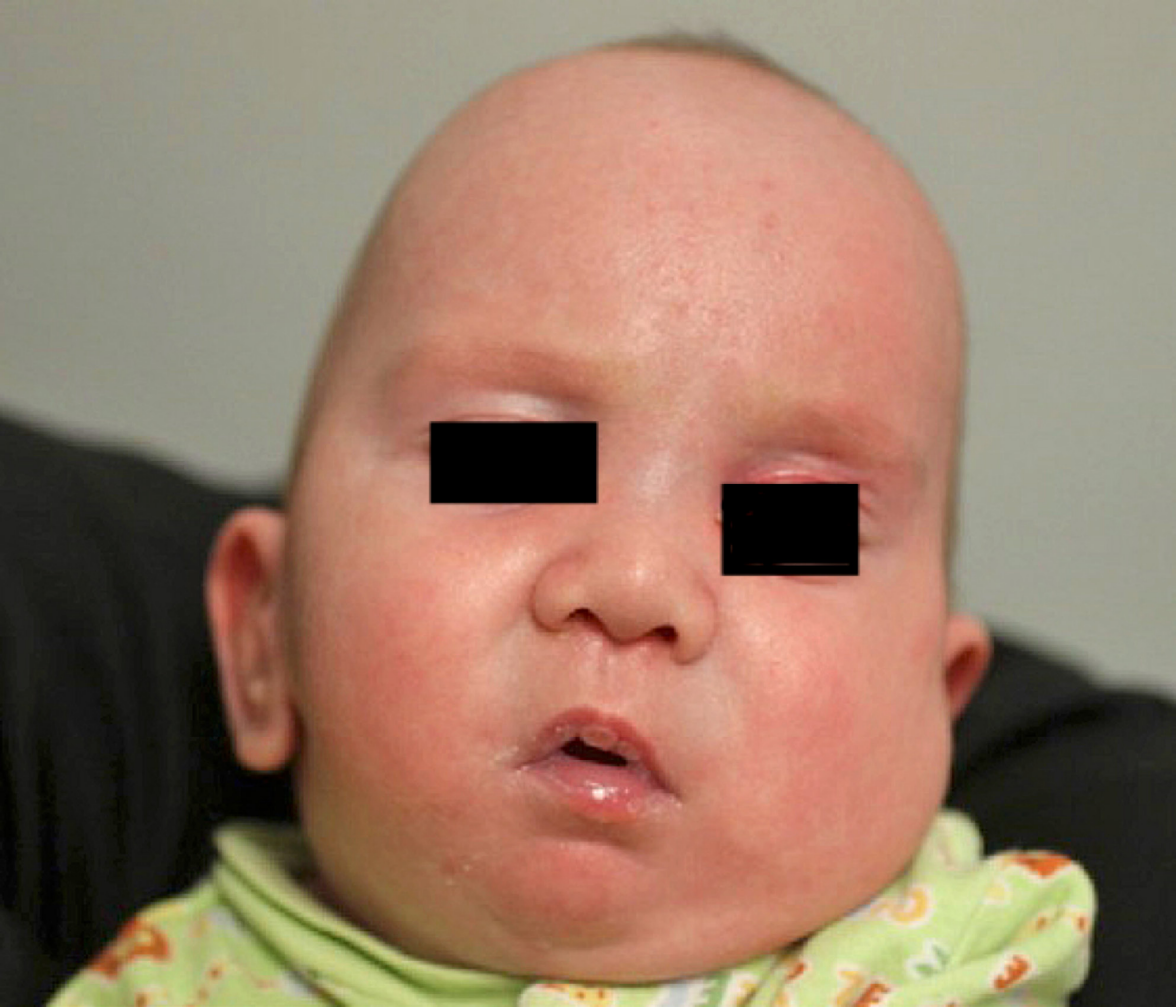
Additional physical findings of a bulbous nose tip, posteriorly rotated low‐set ears, and a small mouth opening.

The specialty access team placed a 24‐gauge intravenous access site in the right forearm of the patient. We administered 30 mcg of glycopyrrolate prior to induction of general anesthesia to dry secretions. Given the patient's prior difficult intubation, an otolaryngology staff was present during the induction of general anesthesia and the intubation procedure. We were prepared in the event rigid bronchoscopy or a surgical airway became necessary. We performed an inhalation mask induction with sevoflurane without respiratory issues. We maintained spontaneous ventilation with the infant in the right lateral position given the improved ventilation in this position. We administered incremental boluses of ketamine to a total of 10 mg. We positioned the infant supine for intubation. We utilized video laryngoscopy with a C‐MAC® miller 0 blade (KARL STORZ). The first intubation attempt failed due to the extreme anterior view and the small mouth opening (Figure 2). Minor repositioning and external laryngeal manipulation by the otolaryngologist resulted in a successful intubation on the second attempt. With the airway secured, we administered rocuronium. We administered sevoflurane for maintenance anesthesia. With the known skeletal anomalies, we did not perform a caudal regional block at the beginning of the case. We administered fentanyl for a short‐acting analgesic. The surgeons placed local anesthesia at the surgical sites at the end of the procedure, and we administered intravenous acetaminophen for analgesia. We reversed the muscle relaxation with sugammadex. We placed a 16 French nasal trumpet in the left nare to optimize ventilation and to reduce airway obstruction. We placed the infant in the right lateral position and extubated only after the patient was fully awake. The infant returned to the neonatal intensive care unit with the nasal trumpet in place. The patient recovered without any issues.

## 3 DISCUSSION

FHUFS was first reported in 1961. It is a unique phenotype consisting of asymmetrical hypoplasia of the femurs in association with abnormal facial characteristics. A diagnosis of FHUFS relies on the physical findings of significant femoral hypoplasia and the presence of at least two of four facial features: up slanting eyes, hypoplastic alae nasi with a broad nasal tip, a long philtrum with a thin upper lip and palate, maxillary asymmetry, an isolated cleft palate, and low‐set poorly formed pinna.^1^ Concurrent findings of Pierre Robin sequence with glossoptosis, cleft palate, and airway obstruction may be present.^3^

Patients with FHUFS have malformations that affect multiple organ systems.^5^ Physical features identified in infants with FHUFS include lumbar spine and pelvic abnormalities, absence or hypoplasia of the fibulae, clubfeet, radioulnar joint anomalies, and genitourinary anomalies such as cryptorchidism, polycystic or absent kidneys, absent uterus and vagina with normal ovaries, and microphallus.^1^, ^7^ Bilobed lungs and various cardiovascular effects such as ventriculoseptal defect, pulmonary stenosis, and truncus arteriousus exist in neonates with FHUFS.^1^, ^8^ Additionally, patients with FHUFS can have central nervous system malformations including encephalocele, brain heterotopia, agenesis of the corpus callosum, and ventriculomegaly.^1^, ^7^

Given the multi‐organ involvement possible in FHUFS, patients regularly present for surgical interventions. These patients might need placement of a gastrostomy tube, orchiopexy, cleft palate repair, and correction of clubfeet. Close attention to hemodynamic monitoring and positioning is imperative. These individuals may have cardiac and renal anomalies along with skeletal malformations. Intraoperatively, the surgeon repeatedly noted the unusual location of anatomic structures in our patient. Neuraxial anesthesia may entail greater risk in individuals with FHUFS with lumbar, sacral, and vertebral anomalies.^9^

This case report describes an anesthetic administered to an infant with FHUFS and Pierre Robin sequence. Both intubation and mask ventilation were important concerns when preparing for the case. Patients with FHUFS can also have challenging intravenous access, and using an ultrasound may facilitate placement. While we secured the airway with video laryngoscopy and external laryngeal manipulation, a fiberoptic scope through a supraglottic airway or the combined use of a fiberoptic scope with video laryngoscopy could be useful to orally intubate a similar patient. If an emergency intervention was necessary, an awake intubation may be required to safely secure the patient's airway. Additionally, it might be beneficial to have otolaryngology staff present in case a rigid bronchoscopy or a surgical airway becomes necessary. Any patient with a known difficult airway for intubation can benefit from similar anesthetic management as described in this case report. Preparation for management with the proper equipment and personnel available is necessary to provide safe anesthetic care of patients with FHUFS.

## AUTHOR CONTRIBUTIONS

**Lauren M. Partyka:** Writing – original draft.

## FUNDING INFORMATION

No disclosures of funding.

## CONFLICT OF INTEREST STATEMENT

No conflict of interests/financial disclosures.

## CONSENT

Written informed consent was obtained from the patient's parents to publish this report in accordance with the journal's patient consent policy.

## Data Availability

There are no external data relevant to this case report.
